# Supplementary material for: A prospective study of mental wellbeing, quality of life, human-animal attachment, and grief among foster caregivers at animal shelters
Source: PLoS One. 2024 May 22;19(5):e0301661. doi: 10.1371/journal.pone.0301661 (PMC11111037; doi:10.1371/journal.pone.0301661)
Supplement: S1 Table — (DOCX) [file pone.0301661.s001.docx]

| **S1 Table.** Standardized factor loadings of DOQOL from confirmatory factor analysis using a maximum likelihood model with bootstrapping including 500 samples. | | | | | | | |
| --- | --- | --- | --- | --- | --- | --- | --- |
|  | **Factor One**  *Emotional QoL* | | **Factor Two**  *Social/Physical QoL* | | **Factor Three**  *Stress/Interference QoL* | | **P** |
| **Survey items** | **Factor loading** | **SE** | **Factor loading** | **SE** | **Factor loading** | **SE** |  |
| Fostering an animal provides me love and affection | 0.82 | 0.05 |  |  |  |  | <0.01 |
| Fostering an animal provides me companionship when I want it | 0.94 | 0.03 |  |  |  |  | <0.01 |
| Fostering an animal provides me emotional support | 0.89 | 0.04 |  |  |  |  | <0.01 |
| Fostering an animal improves the amount of social activities I perform |  |  | 0.78 | 0.08 |  |  | <0.01 |
| Fostering an animal improves my ability to do things for fun outside my home |  |  | 0.92 | 0.10 |  |  | <0.01 |
| Fostering an animal improves my level of physical activity |  |  | 0.67 | 0.10 |  |  | <0.01 |
| Fostering an animal interferes with my other household responsibilities |  |  |  |  | 0.83 | 0.09 | 0.01 |
| Fostering an animal results in damage to my belongings or property |  |  |  |  | 0.61 | 0.11 | <0.01 |
| Fostering an animal interferes with my ability to go on vacation or leave my house |  |  |  |  | 0.81 | 0.08 | <0.01 |
| Fostering an animal increases my level of stress |  |  |  |  | 0.67 | 0.11 | <0.01 |
| SE shows standard error. | | | | | | | |
